# *Andinopanurgus*, a new Andean subgenus of *Protandrena* (Hymenoptera, Andrenidae)

**DOI:** 10.3897/zookeys.126.1676

**Published:** 2011-09-02

**Authors:** Victor H. Gonzalez, Michael S. Engel

**Affiliations:** 1 Department of Ecology & Evolutionary Biology, 1200 Sunnyside Avenue, Haworth Hall, University of Kansas, Lawrence, Kansas 66045, USA; Current address: USDA-ARS Bee Biology & Systematics Laboratory, Utah State University, Logan, Utah 84322-5310, USA; 2 Division of Entomology (Paleoentomology), Natural History Museum, and Department of Ecology & Evolutionary Biology, 1501 Crestline Drive – Suite 140, University of Kansas, Lawrence, Kansas 66045, USA

**Keywords:** Anthophila, Apoidea, Panurginae, *Heterosarus*, *Rhophitulus*, South America, taxonomy

## Abstract

A new subgenus of *Protandrena* Cockerell (Panurginae: Protandrenini) from South America, *Andinopanurgus* Gonzalez & Engel, **subgen. n**., is described and figured for distinctive species of the genus occurring at mid- and high elevations in the Andes from Venezuela to Peru (1100–3400 m). In addition to the distribution, the subgenus is easily distinguished from other subgenera by a unique combination of morphological characters in both sexes, especially in the hidden sterna and genitalia of the male. *Protandrena amyae* **sp. n.**, and *Protandrena femoralis* **sp. n.**, are also described and figured from the Ecuadorian and Peruvian Andes. New geographical records and a key to the species are also provided.

## Introduction

Panurgine bees of the tribe Protandrenini (*sensu* Michener 2007) are restricted to the Western Hemisphere where they are diverse and abundant in temperate areas of the Americas but poorly represented to nearly absent in the tropics. The tribe is relatively small, consisting of about 400 species (Ascher and Pickering 2011) grouped into 11 genera and multiple subgenera (Table 1), some of them, particularly those in the genus *Protandrena* Cockerell, are treated at the generic level (e.g., Ruz 1986; Moure et al. 2008; Ascher and Pickering 2011).

Although depauperate in andrenid bees by comparison to the temperate Andes, during the last decade several species of panurginae have been identified from the tropical Andes, from Bolivia to Venezuela (Gonzalez 2004; Gonzalez and Engel 2004; Gonzalez and Ruz 2007). This is particularly the case for five unusual panurgine species distributed from Venezuela, Colombia, and Ecuador described by Gonzalez and Ruz (2007) in the genus *Protandrena* (*sensu* Michener 2007). These species are morphologically distinctive and, as in other temperate South American *Protandrena* s.l. (e.g., Ruz and Chiappa 2004; Ramos and Melo 2006), they could not be assigned to any of the available subgenera. Although tropical Andean bees are still poorly documented and underrepresented in collections, recent appraisals of museum specimens have revealed a couple of additional new panurgine species belonging to this same group. Although initially regarded as oddities, it is becoming obvious to us that these species form a clear lineage of tropical Andean panurgines, distinct from other subgenera of *Protandrena* s.l. and easily recognized by a unique combination of morphological characters in both sexes, but particularly in the male. Herein we propose a new subgeneric name, *Andinopanurgus* Gonzalez and Engel subgen. n., for these species and describe the two new species from Ecuador and Peru. We also provide new geographical records and a key to species in the hope of drawing these to the attention of melittologists working with the Andean fauna.

**Table 1.** Summary of generic and subgeneric classification of Protandrenini (*sensu* Michener 2007), with the new subgenus included. Those subgenera still recognized at the generic level by some authors are indicated with an asterisk; note that *Neffapis* has been segregated into a separate tribe, Neffapini (Engel 2005). The distribution and approximate number of species are based on Michener (2007), Moure et al. (2008), and Ascher and Pickering (2011). NA = North America; CA = Central America; SA = South America.

Taxa	Species	Distribution
Genus *Anthemurgus* Robertson	1	NA (USA)
Genus *Anthrenoides* Ducke	65	SA (Argentina, Brazil, Chile, Paraguay)
Genus *Chaeturginus* Lucas de Oliveira & Moure	2	SA (Brazil)
Genus *Liphanthus* Reed		
Subgenus *Leptophanthus* Ruz & Toro	9	SA (Argentina, Chile)
Subgenus *Liphanthus* Reed	9	SA (Chile)
Subgenus *Melaliphanthus* Ruz & Toro	3	SA (Chile)
Subgenus *Neoliphanthus* Ruz & Toro	1	SA (Chile)
Subgenus *Pseudoliphanthus* Ruz & Toro	4	SA (Argentina, Chile)
Subgenus *Tricholiphanthus* Ruz & Toro	3	SA (Chile)
Subgenus *Xenoliphanthus* Ruz & Toro	4	SA (Chile)
Genus *Neffapis* Ruz	1	SA (Chile)
Genus *Parapsaenythia* Friese	7	SA (Argentina, Bolivia, Brazil, Paraguay)
Genus *Protandrena* Cockerell		
Subgenus *Andinopanurgus* subgen. n.	7	SA (Colombia, Ecuador, Peru, Venezuela)
Subgenus *Austropanurgus* Toro*	1	SA (Chile)
Subgenus *Heterosarus* Robertson*	41	NA, CA, SA
Subgenus *Metapsaenythia* Timberlake	1	NA (Mexico, USA)
Subgenus *Parasarus* Ruz*	1	SA (Argentina, Chile)
Subgenus *Protandrena* Cockerell*	~50	NA, CA, SA
Subgenus *Pterosarus* Timberlake*	~40	NA, CA (Canada to Guatemala)
Genus *Psaenythia* Gerstaecker	~80	SA (Argentina, Brazil, Chile)
Genus *Pseudopanurgus* Cockerell	33	NA, CA (USA to Costa Rica)
Genus *Rhophitulus* Ducke		
Subgenus *Cephalurgus* Moure & Lucas de Oliveira*	5	SA (Brazil, Paraguay)
Subgenus *Panurgillus* Moure	21	SA (Brazil, Argentina)
Subgenus *Rhophitulus* Ducke	3	SA (Brazil, Argentina)
*Incertae sedis*		
Genus *Stenocolletes* Schrottky	1	SA (Argentina)

## Material and methods

Morphological terminology follows that of Engel (2001) and Michener (2007), while the format for the description generally follows that used by Gonzalez and Ruz (2007). Photomicrographs were prepared using a Nikon D1× digital camera attached to an Infinity K-2 long-distance microscopic lens. Measurements were made with an ocular micrometer attached to an Olympus SZX-12 stereomicroscope. Measurements in descriptions are for the holotype, with values for paratypes in parentheses. The abbreviations F, S, T, OD, and PW are used for antennal flagellomere, metasomal sternum and tergum, and ocellar diameter and puncture width, respectively. Type specimens are deposited in the National Museum of Natural History, Washington, DC, USA (USNM) and the Snow Entomological Collection, Division of Entomology, University of Kansas Natural History Museum, Lawrence, Kansas, USA (SEMC).

## Systematics

### **Tribe Protandrenini Robertson, 1904 Genus **Protandrena** Cockerell, 1896**

#### Andinopanurgus

Gonzalez & Engel subgen. n.

urn:lsid:zoobank.org:act:1F38EF71-936F-4F45-9A56-F84107823BE9

http://species-id.net/wiki/Andinopanurgus

##### Type species.

*Protandrena bachue* Gonzalez & Ruz, 2007.

##### Diagnosis.

**Figures 1–5.**
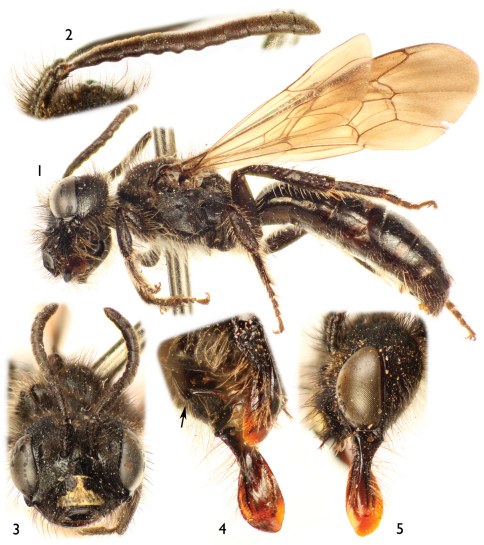
Male of *Protandrena (Andinopanurgus) amyae* Gonzalez and Engel, **sp. n.** **1** Lateral habitus of holotype. **2** Detail of paratype antenna showing crenulations. **3** Facial aspect of holotype. **4** Oblique ventral view of paratype head showing hypostomal tooth (arrow). **5** Lateral aspect of paratype head.

**Figures 6–12.**
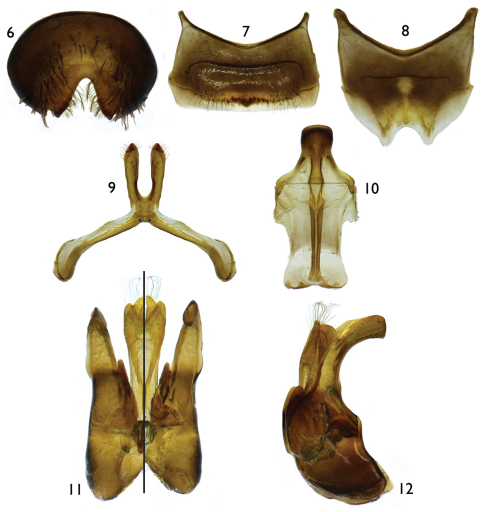
Male terminalia of *Protandrena (Andinopanurgus) amyae* Gonzalez and Engel, **sp. n.** **6** Apical view of tergum VII. **7** Sternum V. **8** Sternum VI **9** Sternum VII. **10** Sternum VIII. **11** Genital capsule (left half is dorsal aspect, right half is ventral aspect). **12** Lateral aspect of genital capsule.

**Figures 13–14.**
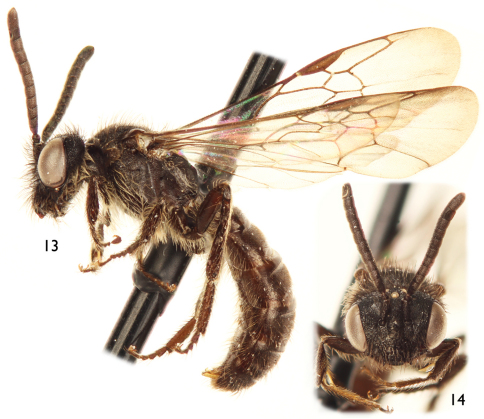
Male of *Protandrena (Andinopanurgus) femoralis* Gonzalez and Engel, **sp. n.** **13** Lateral aspect. **14** Facial aspect.

The new subgenus can be recognized easily by the following combination of characters: body predominantly dark brown to black with reduced yellow maculations; forewing with two submarginal cells (e.g., Figs 1, 13); propodeum glabrous basally; mesoscutum finely punctate; dorsal surface of propodeum longer than metanotum; anterior tentorial pit at outer subantennal sulcus, just above intersection between outer subantennal and epistomal sulci; female metatibial scopa with sparse, mostly simple setae; and male SVI with broad U- or V-shaped midapical emargination (Figs 8, 16); TVII with distal margin straight or medially emarginate (Fig. 6); and gonostylus simple, without apical lobes or projections, without long apical setae, and completely fused to gonocoxite (Figs 11, 12, 19, 20).

##### Description.

*Female*:Small to moderate-sized bees (4–12 mm in length); color mainly dark brown to black, nonmetallic, without yellow maculations except on pronotal lip; integument dull to weakly shiny, distinctly imbricate to granular between punctures, especially on dorsal surface of mesosoma and posterior surface of mesofemur; punctures coarser, denser on head than on meso- and metasoma; pubescence predominantly dark brown to black, short, and sparse; pubescence longer and denser on head and mesosoma; metatibial scopa consisting of sparse, long, mostly simple setae; metasomal terga and sterna, except on apical segments, with distal margins glabrous; SVI with dense patch of branched setae laterally (cf. Gonzalez and Ruz 2007: Figs 41–44). Head broader than long, about as wide as mesosoma; mandible edentate, pointed; labrum with strong ridge bordering glabrous, impunctate basal area; clypeus less than three times broader than long; supraclypeal area usually more distinctly convex than clypeus in profile; lower mesal paraocular area gently convex; anterior tentorial pit at outer subantennal sulcus, just above epistomal sulcus; antennal toruli about at middle of face; antennal scape unmodified, not surpassing lower tangent of median ocellus in repose; antennal flagellum about as long as head width or longer, unmodified or crenulate basally on posterior surface; facial fovea well-marked, elongate; compound eyes subparallel; lower margin of median ocellus coinciding with upper orbital tangent; vertex gently convex; gena about as wide as or slightly wider than compound eye in profile, widest dorsally, narrower ventrally; labiomaxillary complex of moderate length, not distinctly elongate; maxillary palpus with six equally long palpomeres; labial palpus with four palpomeres, first palpomere about as long as combined lengths of remaining palpomeres, second palpomere about as long as third and fourth palpomeres individually; glossa about one-half length of prementum; galeal comb composed of 20 bristles. Pronotal collar rounded, not carinate; dorsal surface of propodeum gently sloping to subhorizontal, longer than metanotum, weakly striate. Forewing with pterostigma more than three times longer than broad, about twice as wide as prestigma, margin basal to vein r-rs diverging from costa, that within marginal cell slightly convex; marginal cell obliquely and broadly truncate at apex, appendiculate, slightly longer than distance from its apex to wing tip; two submarginal cells (i.e., 1rs-m absent), first submarginal cell longer than second; basal vein gently curved to nearly straight; 1m-cu distal to 2Rs (second free abscissa Rs, or first submarginal crossvein *sensu* Michener 2007); 2m-cu basal to 2rs-m (second submarginal crossvein *sensu* Michener 2007); jugal lobe about three-fourths length of vannal lobe; hind wing with second abscissa of M+Cu more than three times length of cu-a; 6–8 distal hamuli. Legs unmodified; mesofemur without well-developed comb on ventral margin basally; mesotibial spur slightly shorter than mesobasitarsus, straight or nearly so, with coarse branches (*sensu* Engel 2009) (cf. Gonzalez and Ruz 2007: Figs 39, 40); metatibia about twice as long as metabasitarsus, keirotrichia on inner surface except on anterior and posterior margins; metabasitibial plate carinate, with semierect, short, stiff setae on disc; metatibial spurs slightly curved apically to nearly straight; metabasitarsus strongly projecting on posterodistal margin; pretarsal claws cleft, inner ramus shorter than outer. Metasomal TII with well-marked lateral fovea; pygidial plate subtriangular, well-defined, medially elevated; SVI with distal margin rounded or truncate.

*Male*: As in female except longer, sparser body pubescence, clypeus often maculate, metabasitibial plate glabrous, and the following: antennal flagellum unmodified (Figs 13, 27) to weakly (Fig. 2) or strongly (Fig. 26) crenulate on posterior surface; compound eyes subparallel to slightly convergent ventrally. Outer surfaces of pro- and mesotibiae apically with small spine; metatibia with posterior marginal carina weakly toothed basally; metabasitarsus with posterodistal margin not distinctly projecting as in female; pretarsal claws symmetrical or with inner ramus shorter than outer. Metasoma usually more elongate than in female, sometimes petiolate; TVII without pygidial plate, distal margin straight or with V-shaped median emargination (Fig. 6); sterna with distal margins straight or convex, except SVI with distinct U- or V-shaped median emargination (Figs 8, 16); SVII with apical lobes attached to small discal area, not distinctly constricted basally, distally retrorse (Figs 9, 17, 30); SVIII longer than broad, midapical projection rather short (about one-half length of disc body), not distinctly constricted basally, broadly rounded apically (Figs 10, 18); genital capsule slightly longer than broad, gonobase absent; gonostylus about as long as penis valves or slightly longer, simple, fully fused to gonocoxite, gently or strongly curved in profile, without long, branched setae on apex; volsella clearly differentiated in medial digitus and lateral cuspis, denticulate, digitus elongate; penis valves simple, narrow, basally fused; penis membranous, bilobed, apically wide, about as long as penis valves (Figs 11, 12, 19, 20).

##### Etymology.

 The new genus-group name is a combination of Andes, referring to the Andean distribution of this group of bees, and *Panurgus*, type genus of the Panurginae. The name is masculine.

##### Included species.

In addition to the type species, *Protandrena bachue* Gonzalez & Ruz, the subgenus includes the following taxa: *Protandrena amyae* sp. n., *Protandrena femoralis* sp. n., *Protandrena guarnensis* Gonzalez & Ruz, *Protandrena maximina* Gonzalez & Ruz, *Protandrena rangeli* Gonzalez & Ruz, and *Protandrena wayruronga* Gonzalez & Ruz.

##### Comments.

The subgenus occurs at mid- and high elevations (1100–3400 m) in the Andes from Venezuela to Peru. Two species groups (one consisting of *Protandrena guarnensis* and *Protandrena femoralis*, the other including the remaining species) can be recognized within *Andinopanurgus* by the characters indicated in the key to species (*infra*) (Table 2).

The general habitus of *Andinopanurgus* as well as the shape of the male sixth and seventh sterna suggest species of *Rhophitulus* Ducke and *Heterosarus* Robertson but the propodeum is basally pubescent in *Rhophitulus* and, in both taxa, TVII is gently or strongly projected medially on the distal margin, SVII has lobes with much broader apex, and the gonostylus has long branched setae apically and is partially fused to the gonocoxite, at least ventrally. In addition, *Andinopanurgus* lacks the distinctive dorsal remnant of the gonobase of *Rhophitulus*. The rather narrow and distally retrorse apical lobes of SVII of *Andinopanurgus* (Figs 9, 17) resemble those of *Protandrena* s.str. and *Metapsaenythia* Timberlake, but the apex of these lobes lack the distinctive spatulate setae present in the latter. *Metapsaenythia* has also a propodeum basally pubescent and a metasoma that is frequently red. If future studies demonstrate that *Protandrena* s.l. is paraphylectic, perhaps some of its subgenera, including *Andinopanurgus*, may well be recognized at the generic level.

**Table 2.** Summary of currently included species in *Andinopanurgus* with information on the known sexes, distribution, and some morphological characters. Plus (+) and dash (–) symbols indicate presence and absence of a particular character, ? = unknown.

Taxon	Sexes known	Distribution	Elevation (m.s.l.)	Male		Female
				Antennal flagellum	Spines of SV	Antennal flagellum
“bachue species group”						
*Protandrena amyae* sp. n.	♂	Ecuador: Napo	2438	weakly crenulate	+	?
*Protandrena bachue* Gonzalez & Ruz	♂♀	Colombia: Boyacá, Cundinamarca	2830–3380	strongly crenulate	+	weakly crenulate
*Protandrena maximina* Gonzalez & Ruz	♀	Venezuela: Mérida	2360	?	?	unmodified
*Protandrena rangeli* Gonzalez & Ruz	♂♀	Colombia: Boyacá, Cundinamarca	2600–2830	unmodified	+	unmodified
*Protandrena wayruronga* Gonzalez & Ruz	♂	Ecuador: Napo, Pichincha	3150	strongly crenulate	+	?
“*guarnensis* species group”						
*Protandrena guarnensis* Gonzalez & Ruz	♂♀	Colombia: Antioquia	2000	unmodified	–	unmodified
*Protandrena femoralis* sp. n.	♂♀	Peru: Pasco	1100–1780	unmodified	–	unmodified

#### Protandrena (Andinopanurgus) amyae

Gonzalez & Engel sp. n.

urn:lsid:zoobank.org:act:BBCDA150-6DF7-4F65-824C-638DA480DF87

http://species-id.net/wiki/Protandrena_(Andinopanurgus)_amyae

Figs 1 12

##### Holotype.

 ♂ (Fig. 1), Ecuador: Napo. Past. Road from Baeza to Papallacta, km. 188, 13-IV-1977 [13 April 1977], Elaine R. Hodges (USNM).

##### Paratype.

 ♂, Ecuador: Napo, Baeza (22 Kms. W.), 15 May 1975, elev. 8000ft. / Collected by sweeping net above damp road bed / Collected by Ashley B. Gurney (SEMC).

##### Diagnosis.

 The male of this species can easily be recognized by the antennal flagellum weakly crenulate on the posterior surface (Fig. 2), the mandible distinctly broad apically (Figs 4, 5), and the posterior hypostomal carina strongly projecting into a tooth (Fig. 4).

##### Description.

*Male*: Body length 8.70 mm (8.50 mm); forewing length 6.50 mm (6.70 mm); head width 2.40 mm (2.48 mm). Head 1.4× wider than long; inner orbits of compound eyes subparallel (Fig. 3); intertorular distance 1.7× OD, 0.7× length of torulorbital distance; torulus diameter equal to OD; ocellocular distance 3.4× OD, 2.0× greater than ocelloccipital distance; interocellar distance 1.3× OD; compound eye 1.8× longer than wide; clypeus 2.6× broader than long, projecting about 0.4× compound eye width in lateral view; gena 1.2× broader than compound eye in profile; supraclypeal area, just below inferior torular tangent, distinctly protuberant; frontal line elevated just above antennal toruli to one-half distance between antennal toruli and median ocellus, ending at that point; inner subantennal sulcus about 0.7× length of outer subantennal sulcus; facial fovea 1.7× longer than broad, about one-half length of scape; scape 2.1× longer than broad, antennal flagellum slightly longer than head width, F1–F6 weakly crenulate on posterior surface, not forming deep concavity between flagellomeres (Fig. 2); pedicel about one-third length of F1, about as long as broad, F1 1.8× longer than broad, about 1.5× longer than F2 and F3 individually, remaining flagellomeres about as long as broad, except last flagellomere longer than broad; mandible distinctly broad apically (Figs 4, 5); posterior hypostomal carina strongly projecting into a tooth (Fig. 4). Forewing pterostigma 4.0× longer than broad; prestigma 3.1× longer than broad (prestigma width measured to its margin). Mesosoma slightly narrower than head width; mesoscutum 1.3× wider than long, 2.3× longer than mesoscutellum, 4.5× longer than metanotum; propodeum with basal part about three-fourths of mesoscutellum length in dorsal view; protibial spur with apical portion of rachis long, about three-fourths of malus length, with distinct row of 10 elongate branches (not including apical portion of rachis); mesotibial spur gently curved apically, with coarse branches, less than one-half of mesobasitarsus length; metatibia with posterior marginal carina weakly toothed on upper two-thirds; metatibial spurs slightly curved apically, inner spur slightly longer than outer; pretarsal claws cleft, inner ramus slightly shorter than the outer. Lateral fovea of TII ellipsoid, about 2.0× longer than broad; TVII with V-shaped median emargination on distal margin (Fig. 6); SV–SVIII, and genital capsule as in figures 7–12.

Color dark brown to black, except apex of mandible reddish brown and clypeus with yellow maculation as in figure 3. Wing membranes brownish, veins and pterostigma dark brown.

Head with sparse, long (2.5–3.0× OD), semierect, poorly-branched, black setae except brownish setae on condylar and outer grooves of mandible, gena posteriorly, and hypostomal area; scape with long setae, 2× as long as maximum scape diameter. Pronotum with short (0.5–1.0× OD), dense, brownish setae along dorsal margin and pronotal lobe; mesoscutum, mesoscutellum, and metanotum with two types of setae: sparse, long (2.5–3.0× OD), erect, poorly-branched, black setae, and dense, short (0.5× OD), brownish setae; mesepisternum and lateral and posterior areas of propodeum with mostly sparse, long (2.5–3.0× OD), erect, branched, brownish setae; legs with setae mostly brownish, longer and denser on coxae, trochanters, and profemur. Metasoma with terga mostly bare, with minute (≤ 0.3× OD), semierect, sparse ferruginous setae on discs, laterally with denser and longer setae; TVI with long (2× OD), semierect, dark brown setae on disc, setae denser on TVII; sterna with sparse, short (1× OD), semierect setae, denser and longer on sides of each sternum.

Outer surface of mandible and basal area of labrum smooth and shiny, impunctate; clypeus with sparse (1–1.5× PW), faint punctures, integument between punctures imbricate; supraclypeal area with scattered punctures laterally, weakly imbricate, shinier than on clypeus medially; subantennal area and inferior paraocular area with punctures separated by a puncture width or less, integument strongly imbricate to nearly granular (as on remainder of face); remaining areas of face with coarse punctures, contiguous, smaller than on clypeus; gena strongly imbricate with faint punctures. Mesoscutum, mesoscutellum, and metanotum with small, dense punctures (≤ 1× PW), integument granular between punctures; mesepisternum strongly imbricate with scattered (1–2.0× PW), faint punctures, punctures coarser and denser dorsally; metepisternum transversely weakly striate near wing base, otherwise strongly imbricate. Propodeum strongly imbricate with fine and weak striae basally, lateral and posterior surfaces with faint, scattered punctures. Metasomal terga and sterna shiny, weakly imbricate with minute, scattered (2–3.0× PW) punctures on discs, punctures coarser and denser on TVII; distal margins of terga shiny, weakly imbricate, impunctate except on TVII.

*Female*: Unknown.

##### Etymology.

 The specific epithet is a matronym honoring Mrs. Amy Comfort de Gonzalez, loving and supporting wife of the senior author.

#### Protandrena (Andinopanurgus) femoralis

Gonzalez & Engel sp. n.

urn:lsid:zoobank.org:act:BB7E3450-892C-4DBA-8EBA-ADABB1135D4F

http://species-id.net/wiki/Protandrena_(Andinopanurgus)_femoralis

Figs 13 [Fig F4] 25

##### Holotype.

 ♂, Peru: Pasco Dept. [Departamento] San Miguel Eneñas, NW Villa Rica-Puerto Bermudas Rd., 1780 m, 10°44'0"S, 75°11'54"W, 16 Oct 1999; R. Brooks, PERU1B99 037, ex: yellow composite / SM0149359, KUNHM-ENT [barcode label] (SEMC).

##### Paratypes.

 (*n* = 3♀♀, 12♂♂) 2♂♂ with same date as the holotype; 1♂, Pasco Dept. San Juan, Villa Rica-Puerto Bermudas Rd., Rio Cacazu, 1100 m, 10°39'12"S, 75°6'54"W, 16 Oct 1999; R. Brooks, PERU1B99 034A, ex: on flowering tree; 3♀♀, 9♂♂, Pasco Dept. Villa Rica Rd., 1475 m, 10°47'6"S, 75°18'54"W, 15 Oct 1999; R. Brooks, D. Brzoska, PERU1B99 030 (SEMC).

##### Diagnosis.

 Both sexes of *Protandrena femoralis* are most similar to *Protandrena guarnensis* from northwestern Colombia in their small body size (4.5–6.0 mm in body length), F1 about as long as F2, absence of maculations on the male clypeus, male TVII with a straight distal margin, male SV without spines on the midapical margin, and general shape of the genitalia and hidden sterna. The male can easily be separated by the structure of SVII, which has broader apical lobes (cf. Figs 17 and 30), and the gonostylus, which is more robust in profile than that of *Protandrena guarnensis*, about the same width across its length, and basally not protuberant on the medial margin in dorsal view (Figs 19, 20). The female can be recognized by the posterior surface of the mesofemur and anterior and posterior surfaces of the metafemur distinctly depressed (Figs 23–25). In *Protandrena guarnensis* the apical lobes of SVII are narrow, parallel-sided, with the retrorse section of the apex comma-shaped (Fig. 30); the gonostylus is slender in profile, slightly tapering towards the apex, and strongly protuberant basally on the medial margin in dorsal view (cf. Gonzalez and Ruz 2007: Figs 28, 32). The meso- and metafemora of the female of *Protandrena guarnensis* are unmodified, not distinctly depressed.

##### Description.

**Figures 15–20. F4:**
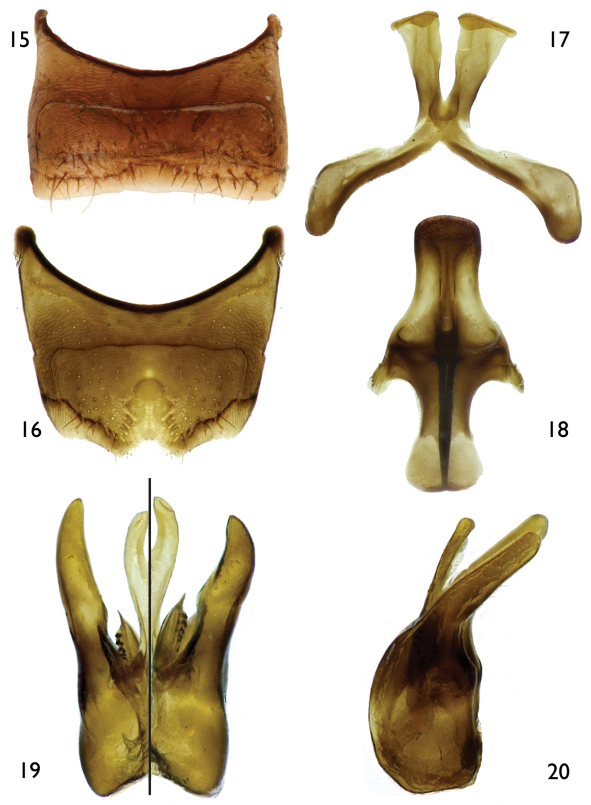
Male terminalia of *Protandrena (Andinopanurgus) femoralis* Gonzalez and Engel, **sp. n.** **15** Sternum V. **16** Sternum VI. **17** Sternum VII. **18** Sternum VIII. **19** Genital capsule (left half is dorsal aspect, right half is ventral aspect). **20** Lateral aspect of genital capsule.

*Male*: Body length 5.0 mm (4.73–5.33 mm); forewing length 4.47 mm (4.47–4.60 mm); head width 1.50 mm (1.50–1.60 mm). Head 1.4× wider than long; inner orbits of compound eyes converging below (Figs 14); intertorular distance 2.3× OD, 0.9× length of torulorbital distance; torulus diameter equal to OD; ocellocular distance 3.4× OD, 2.8× greater than ocelloccipital distance; interocellar distance 1.3× OD; compound eye 1.8× longer than wide; clypeus 2.4× broader than long, projecting about 0.3× compound eye width in lateral view; gena 0.8× width of compound eye in profile; supraclypeal area, just below inferior torular tangent, distinctly protuberant; frontal line weakly elevated just above antennal toruli to one-half distance between antennal toruli and median ocellus, ending at that point; inner subantennal sulcus about 0.7× length of outer subantennal sulcus; facial fovea about 2.0× longer than broad, 0.4× length of scape; scape 2.1× longer than broad, antennal flagellum unmodified, slightly longer than head width (Figs 13–14); pedicel slightly shorter than F1, about as long as broad, F1 about as long as broad, subequal to F2 and F3 individually, remaining flagellomeres about as long as broad, except last flagellomere longer than broad; mandible pointed. Forewing prestigma 3.2× longer than broad (prestigma width measured to its margin); pterostigma 3.6× longer than broad. Mesosoma narrower than head width; mesoscutum 1.3× wider than long, 2.7× longer than mesoscutellum, 5.7× longer than metanotum; propodeum with basal part about three-fourths of mesoscutellum length in dorsal view; protibial spur with apical portion of rachis long, about one-half length of malus, with distinct row of about 10 elongate branches (not including apical portion of rachis); mesotibial spur straight or nearly so, with coarse branches, slightly more than one-half mesobasitarsus length; metatibia with posterior marginal carina weakly toothed on upper third; metatibial spurs of similar length, slightly curved apically; pretarsal claws with rami of similar length. Lateral fovea of TII elongate, about 4.0× longer than broad; TVII with distal margin straight or nearly so; SV–SVIII, and genital capsule as in figures 15–20.

Color dark reddish brown to black, without yellow maculations. Wing membranes subhyaline, slightly brownish, veins and pterostigma dark brown.

Head with sparse, long (2.5–3.0× OD), semierect, poorly-branched, black setae except brownish setae on condylar and outer grooves of mandible, gena posteriorly, and hypostomal area; scape with long setae, 2× as long as maximum scape diameter. Pronotum with short (0.5–1.0× OD), dense, brownish setae along dorsal margin and pronotal lobe; mesoscutum and mesoscutellum with two types of dark brown setae: sparse, long (1.5–2.0× OD), erect, poorly-branched setae, and short (0.5–1.0× OD), slightly denser setae; metanotum with short setae as on mesoscutellum; mesepisternum and lateral and posterior areas of propodeum with very sparse, long (1.5–2.0× OD), erect, branched, brownish setae; legs with setae mostly brownish, longer and denser on coxae, trochanters, and profemur. Metasomal terga with minute (≤ 0.3× OD), semierect, dense ferruginous setae on discs, laterally with denser and longer setae; TVI with long (1.5–2× OD), semierect, dark brown setae on disc, setae denser on TVII; sterna with sparse, short, semierect setae (1.5× OD) on discs, denser and longer laterally; preapical margin of SIV with few, semierect thick setae, each seta consisting of short rachis with three or four long branches, resembling scales or bundles of several setae at low magnifications.

Outer surface of mandible and basal area of labrum smooth and shiny, impunctate; clypeus with sparse (1–1.5× PW), faint punctures, integument between punctures weakly imbricate basally, becoming nearly smooth and shiny toward apex; supraclypeal area with scattered punctures laterally, weakly imbricate, medially shiny as on clypeus; remaining areas of face with coarse punctures separated by a puncture width or less, integument strongly imbricate to nearly granular, punctures becoming weaker and sparser on vertex; gena strongly imbricate with faint punctures. Mesoscutum, mesoscutellum, and metanotum with small, sparse punctures, integument granular between punctures; mesepisternum strongly imbricate with scattered (1–2.0× PW), faint punctures, punctures coarser and denser dorsally; metepisternum transversely weakly striate near wing base, otherwise strongly imbricate. Propodeum strongly imbricate with few fine, weak striae basally (barely visible), lateral and posterior surfaces with faint, scattered punctures. Metasomal terga and sterna shiny, lineolate-imbricate with minute punctures separated by about two puncture widths on discs, punctures coarser and denser on TVII, sparser on sterna; distal margins of terga shiny, weakly imbricate, impunctate except on TVII.

**Figures 21–25.**
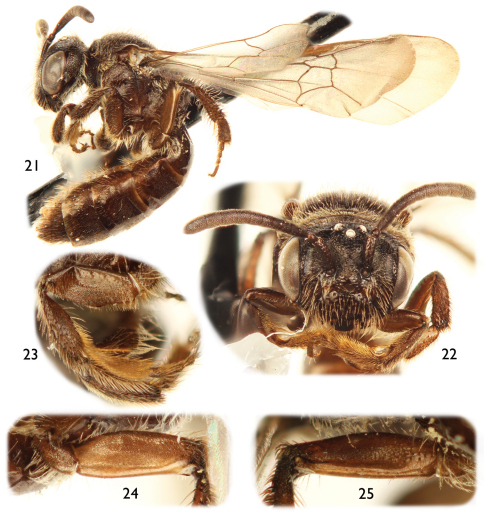
Female of *Protandrena (Andinopanurgus) femoralis* Gonzalez and Engel, **sp. n.** **21** Lateral habitus. **22** Facial aspect. **23** Posterior surface of mesofemur. **24** Anterior surface of metafemur. **25** Posterior surface of metafemur.

*Female*: As in male except shorter body pubescence, lighter and shinier integument (Fig. 21), and the following: Body length 5.53–5.73 mm; forewing length 4.87–5.0 mm; head width 1.60–1.67 mm. Inner orbits of compound eyes subparallel (Fig. 22); intertorular distance 2.6× OD, about as long as torulorbital distance; torulus diameter subequal to OD; ocellocular distance 3.5× OD, 2.5× greater than ocelloccipital distance; interocellar distance 1.6× OD; compound eye 2.1× longer than wide; clypeus 2.9× broader than long, projecting about 0.4× compound eye width in lateral view; supraclypeal area gently convex, not distinctly protuberant medially; facial fovea 3.3× times longer than broad, 0.7× length of scape; scape 2.7× longer than broad, antennal flagellum about as long as head width. Forewing prestigma 5.0× longer than broad (prestigma width measured to its margin); pterostigma 4.5× longer than broad. Mesoscutum about 5.0× longer than metanotum; protibial spur with apical portion of rachis about three-fourths length of malus, with about five branches (not including apical portion of rachis); mesotibial spur about 0.7× mesobasitarsus length; mesofemur with posterior surface and metafemur with anterior and posterior surfaces distinctly depressed (Figs 23–25); inner metatibial spur slightly shorter than outer; pretarsal claws with inner ramus shorter than the outer. Lateral fovea of TII about 3.0× longer than broad.

##### Etymology.

 The specific epithet refers to the distinctly depressed meso- and metafemora of the female of the species.

#### Protandrena (Andinopanurgus) wayruronga

Gonzalez & Ruz

http://species-id.net/wiki/Protandrena_(Andinopanurgus)_wayruronga

Protandrena wayruronga Gonzalez and Ruz, 2007: 400 [♂].
Rhophitulus wayruronga (Gonzalez and Ruz); Ascher and Pickering 2011 [unjustified transfer].

##### New record.

 1♂, Ecuador, Pich. [Pichincha], Quito (48 KmS), 6 May 1975, Ashley Gurney (USNM).

##### Comments.

 This species was previously known from the male holotype collected in Papallacta, Napo (Ecuador). Gonzalez and Ruz (2007) tentatively identified a single specimen from Cauca, Colombia, as the female of this species. The putative female is very similar to another one from Valle, Colombia, except in the length of the apex of the protibial spur; in the specimen from Cauca the apex is long, about one-half of the malus length, whereas in the female from Valle it is short, about one-third of the malus length, as it is in the holotype. Such a difference in both females suggests that they might not be conspecific, although we do not know how variable this character is within and among species. The complete label data of the female from Valle are as follows: “Colombia: Valle, Tenerife, Paramo at 12,000’ R. E. Dietz, Sept. 15, 1970” (USNM).

### Key to Subgenera of Protandrena (modified from Michener 2007)

**Males**

**Table:** 

1	Forewing with three submarginal cells (occasional individuals have only two) (North and Central America)	*Protandrena* (*Protandrena* s. str.)
–	Forewing with two submarginal cells	**2**
2(1)	Propodeal triangle basally pubescent (metasoma often red or largely so) (Nearctic)	*Protandrena (Metapsaenythia)*
–	Propodeal triangle basally glabrous	**3**
3(2)	Metatibial spurs strongly curved at apices; first submarginal cell on posterior margin shorter than second (Chile)	*Protandrena (Austropanurgus)*
–	Metatibial spurs or at least one of them slightly curved or almost straight; first submarginal cell on posterior margin about as long as or longer than second	**4**
4(3)	Gonostylus less than one-third as long as gonocoxite; SVI scarcely notched apically (face black) (South America)	*Protandrena (Parasarus)*
–	Gonostylus over one-half as long as gonocoxite; SVI with deep midapical notch or slit	**5**
5(4)	Mesoscutum with punctures well marked, many of them separated by spaces larger than their diameters; SVI with midapical emargination narrow, deep (North and Central America)	*Protandrena (Pterosarus)*
–	Mesoscutum with punctures very small, homogeneous, commonly dense; SVI midapical emargination U- or V-shaped	**6**
6(5)	TVII gently or strongly projected medially on distal margin; gonostylus with long branched setae apically, partially fused to gonocoxite, at least ventrally	*Protandrena (Heterosarus)*
–	TVII not projecting medially on distal margin, straight or with V-shaped median emargination (Fig. 6); gonostylus without long, branched setae on apex, fully fused to gonocoxite (Figs 11, 12, 19, 20)	*Protandrena (Andinopanurgus)*

**Females**

**Table:** 

1	Forewing with three submarginal cells (only two in occasional individuals) (North and Central America)	*Protandrena* (*Protandrena* s. str.)
–	Forewing with two submarginal cells	**2**
2(1)	Tibial scopa of rather long, abundant setae with clearly visible branches (North and Central America)	*Protandrena (Pterosarus)*
–	Tibial scopa of sparser setae that lack branches, or some of them with few, minute branches	**3**
3(2)	Propodeal triangle basally pubescent (metasoma often largely red) (Nearctic)	*Protandrena (Metapsaenythia)*
–	Propodeal triangle basally glabrous	**4**
4(3)	Metatibial spurs strongly curved at apices; anterior tentorial pit at intersection between outer subantennal and epistomal sulci	**5**
–	Metatibial spurs not strongly curved at apices; anterior tentorial pit not at intersection between outer subantennal and epistomal sulci, just below or above intersection	**6**
5(4)	First submarginal cell on posterior margin shorter than second; face with yellow areas (Chile)	*Protandrena (Austropanurgus)*
–	First submarginal cell on posterior margin longer than second; face black (South America)	*Protandrena (Parasarus)*
6(4)	Anterior tentorial pit in epistomal sulcus slightly to distinctly below intersection between outer subantennal and epistomal sulci; propodeum usually with dorsal surface at most as long as metanotum	*Protandrena (Heterosarus)*
–	Anterior tentorial pit at outer subantennal sulcus, just above intersection between outer subantennal and epistomal sulci; propodeum with dorsal surface longer than metanotum	*Protandrena (Andinopanurgus)*

### Key to species of Andinopanurgus

**Males**

Note that the male of *Protandrena maximina* is unknown.

**Table:** 

1	Clypeus without cream or yellow maculations; F1 short, about as long as F2; face and disc of mesoscutum weakly shiny; SV without spines on midapical margin, with fringe of normal, minutely-branched setae; TVII with distal margin straight, not medially emarginate	**2**
–	Clypeus with cream or yellow maculations; F1 distinctly longer than F2; face and disc of mesoscutum dull; SV with distinctly stout, short spines on midapical margin (Figs 7, 28, 29); TVII with V-shaped median emargination on distal margin	**3**
2(1)	SVII with apical lobes narrow, parallel-sided, retrorse section of apex comma-shaped (Fig. 30); gonostylus slender in profile, slightly tapering towards apex, basally strongly protuberant on medial margin in dorsal view (Colombia: Antioquia)	*Protandrena guarnensis* Gonzalez & Ruz
–	SVII with apical lobes not parallel-sided, much broader apically (apex about twice as broad as base), retrorse section of apex not comma-shaped (Fig. 17); gonostylus more robust in profile, about same width across its length, basally not protuberant on medial margin in dorsal view (Peru)	*Protandrena femoralis* sp. n.
3(1)	Antennal flagellum weakly (Fig. 2) or strongly (Fig. 26) crenulate on posterior surface; SV with more than four spines on midapical margin; larger bees (body length 7.9–11.8 mm)	**4**
–	Antennal flagellum unmodified, not crenulate on posterior surface (Fig. 27); SV with a row of four spines on midapical margin; small bees (body length 5.7–6.1 mm) (Colombia: Boyacá, Cundinamarca)	*Protandrena rangeli *Gonzalez & Ruz
4(3)	Antennal flagellum strongly crenulate on posterior surface, with deep concavity between flagellomeres (Fig. 26); mandible not distinctly broad apically; posterior hypostomal carina unmodified, without a tooth; protibial spur with apex of rachis very short (less than one-third of malus length), with less than five elongate branches (not including apical portion of rachis); SIII–SV with distal margins distinctly convex; SV with midapical row of spines medially projecting (Figs 28, 29)	**5**
–	Antennal flagellum weakly crenulate on posterior surface, without deep concavity between flagellomeres (Fig. 2); mandible distinctly broad apically (Figs 4, 5); posterior hypostomal carina with strong tooth (Fig. 4); protibial spur with apex long, about three-fourths of malus length, with a distinct row of 10 elongate branches (not including apical portion of rachis); SIII–SIV with distal margins gently convex; SV with midapical row of spines straight, not medially projecting (Fig. 7) (Ecuador: Napo)	*Protandrena amyae* sp. n.
5(4)	F8 and F9 crenulate; SV midapical row of spines of unequal sizes, distal two spines distinctly longer (Fig. 29) (Ecuador: Quito, Napo)	*Protandrena wayruronga* Gonzalez & Ruz
–	F8 and F9 unmodified, not crenulate (Fig. 26); SV with midapical row of spines of about same size, without two distinctly long spines distally (Fig. 28) (Colombia: Boyacá, Cundinamarca)	*Protandrena bachue* Gonzalez & Ruz

**Females**

Note that the females of *Protandrena amyae* and *Protandrena wayruronga* are unknown. However, given that the male of these species have crenulate antennal flagella they likely should run to *Protandrena bachue* in the key.

**Table:** 

1	Antennal flagellum unmodified, not crenulate	**2**
–	Antennal flagellum modified, weakly crenulate on posterior surface of F1–F5 (Colombia: Boyacá, Cundinamarca)	*Protandrena bachue* Gonzalez & Ruz
2(1)	F1 about as long as F2; discs of mesoscutum and mesoscutellum shiny, weakly imbricate between punctures; metatibia with brownish to whitish scopal setae	**3**
–	F1 distinctly longer than F2; discs of mesoscutum and mesoscutellum dull, strongly imbricate between punctures; metatibia with dark brown to black scopal setae	**4**
3(2)	Mesofemur with posterior surface and metafemur with anterior and posterior surfaces distinctly depressed (Figs 23–25) (Peru)	*Protandrena femoralis* sp. n.
–	Meso- and metafemora unmodified, not distinctly depressed (Colombia: Antioquia)	*Protandrena guarnensis* Gonzalez & Ruz
4(2)	Small bees (head width 1.6–1.7 mm; body length 5.6–6.3 mm) (Colombia: Boyacá, Cundinamarca)	*Protandrena rangeli* Gonzalez & Ruz
–	Larger bees (head width 1.9–2.1 mm; body length 7.8 mm) (Venezuela)	*Protandrena maximina* Gonzalez & Ruz


**Figures 26–30.**
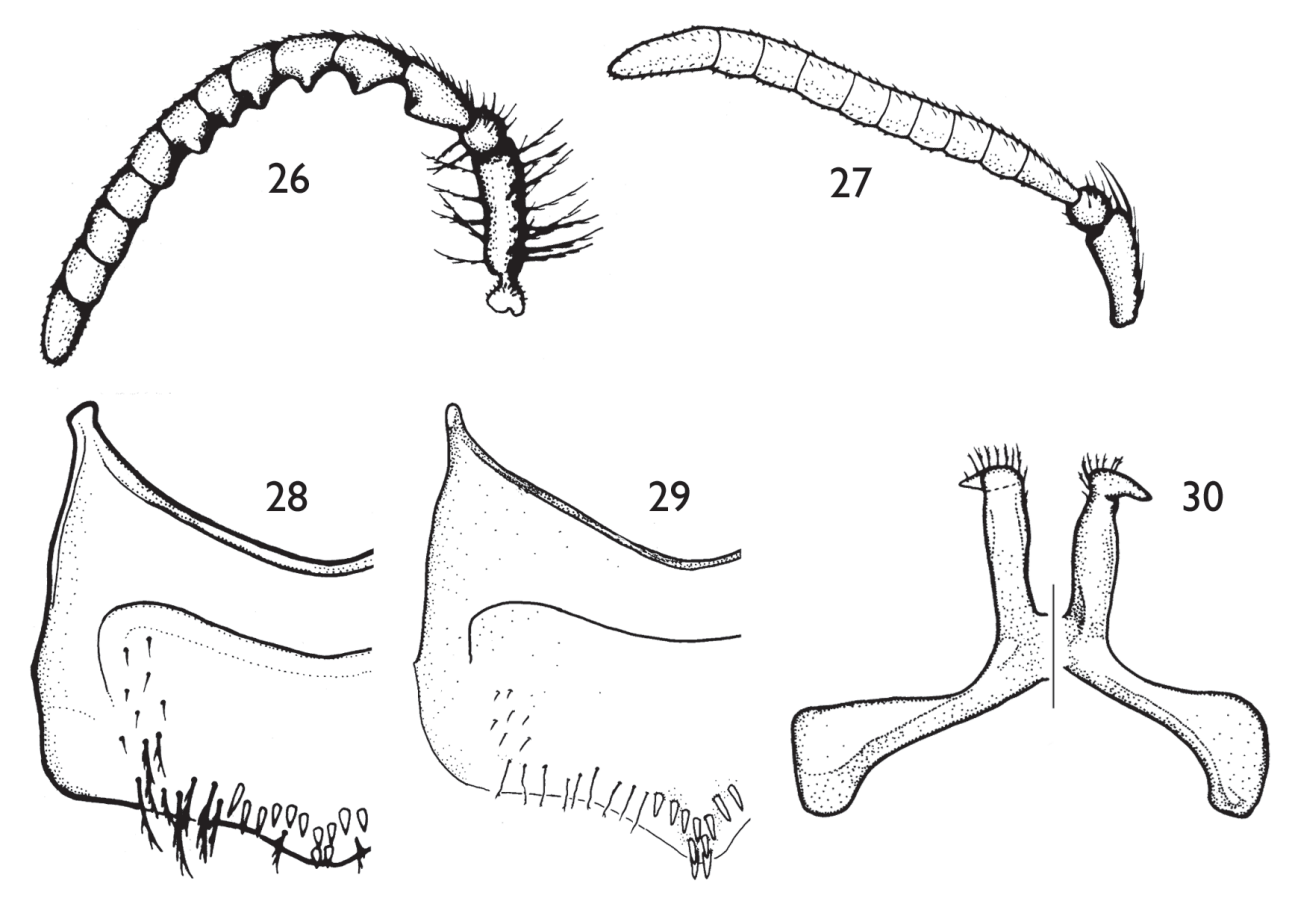
Representative features of *Andinopanurgus* species (from Gonzalez and Ruz 2007). **26.** Male antenna of *Protandrena (Andinopanurgus) bachue* Gonzalez and Ruz. **27** Male antenna of *Protandrena (Andinopanurgus) rangeli* Gonzalez and Ruz. **28** Sternum V of *Protandrena (Andinopanurgus) bachue*. **29** Sternum V of *Protandrena (Andinopanurgus) wayruronga* Gonzalez and Ruz. **30** Sternum VII of *Protandrena (Andinopanurgus) guarnensis* Gonzalez and Ruz.

## Supplementary Material

XML Treatment for Andinopanurgus

XML Treatment for Protandrena (Andinopanurgus) amyae

XML Treatment for Protandrena (Andinopanurgus) femoralis

XML Treatment for Protandrena (Andinopanurgus) wayruronga
